# Solitary left axillary lymph node metastasis after curative resection of carcinoma at the colostomy site: a case report

**DOI:** 10.1186/s40792-016-0229-3

**Published:** 2016-09-21

**Authors:** Ken Imaizumi, Shigenori Homma, Tadashi Yoshida, Tatsushi Shimokuni, Hideyasu Sakihama, Norihiko Takahashi, Hideki Kawamura, Emi Takakuwa, Akinobu Taketomi

**Affiliations:** 1 Department of Gastroenterological Surgery I, Hokkaido University Graduate School of Medicine, North 15, West 7, Kita-ku, Sapporo, Hokkaido 060-8638 Japan; 2 Surgical Center, Hokkaido University Hospital, Sapporo, Hokkaido Japan; 3 Department of Surgical Pathology, Hokkaido University Hospital, Sapporo, Hokkaido Japan

**Keywords:** Colon cancer, Axillary lymph node metastasis, Colostomy site, Pagetoid spread, Adjuvant chemotherapy

## Abstract

**Background:**

The incidence of axillary lymph node metastasis (ALNM) of colon cancer is very low, and there have been only a few reports of solitary ALNM. Neither the mechanism involved in solitary colon cancer ALNM nor the proper treatment has been elucidated. We encountered a case of solitary left ALNM after curative resection of carcinoma at the colostomy site.

**Case presentation:**

A 53-year-old man underwent a Hartmann’s operation for Hirschsprung disease during his adolescence. He complained of a mass of the descending colon and was diagnosed with colon cancer at the colostomy site with pagetoid spread to the adjacent skin. The cancer at the stoma site was resected, and a transverse colostomy was performed. Nine years later, his carbohydrate antigen (CA) 19-9 level was high during a health screening. On physical examination, adenopathy was palpated in the left axilla. Computed tomography (CT) demonstrated a lymph node in the left axillary fossa that was 33 mm in diameter, and ^18^F-fluorodeoxyglucose positron emission tomography/CT showed high uptake in the lesion. We performed a curative resection of the left axillary lymph node. The lesion was pathologically diagnosed as left ALNM originating from the adenocarcinoma at the colostomy site. After lymph node resection, his serum CA19-9 level decreased compared to that observed at baseline. He has been receiving adjuvant chemotherapy (capecitabine plus oxaliplatin) without recurrence for 5 months after lymph node resection.

**Conclusions:**

The present case report shows that carcinoma at the colostomy site with pagetoid spread can metastasize to the axillary lymph nodes through superficial abdominal lymphatic pathways, and surgical resection followed by adjuvant chemotherapy may be a potent strategy to treat solitary colon cancer ALNM.

## Background

Despite curative resection of colon cancer, some patients develop metastasis in the liver, lungs, bone, and lymph nodes [1, 2]. Among these metastases, solitary axillary lymph node metastasis (ALNM) after curative resection of a primary lesion is extremely rare. The mechanism involved in solitary colon cancer ALNM and treatment for the disease have not yet been elucidated. Here, we report a case of solitary left ALNM of descending colon cancer at the stoma site.

## Case presentation

**Fig. 1:**
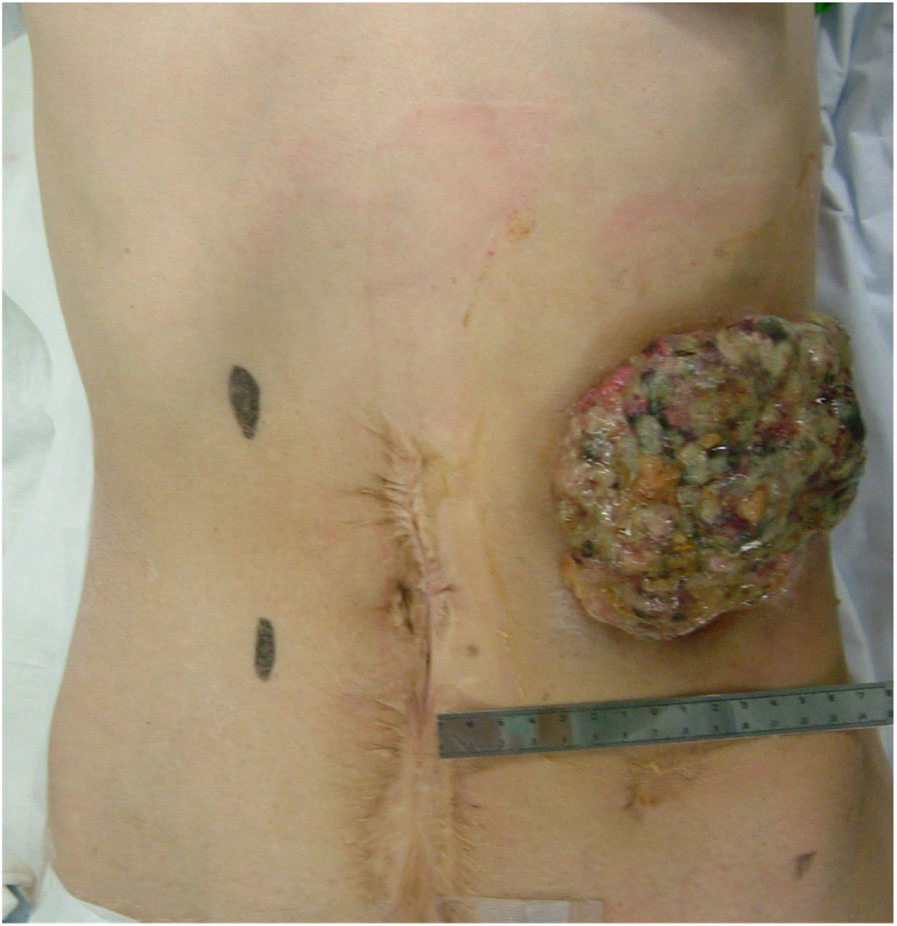
A tumor, 10 cm in diameter, was located at the colostomy site

**Fig. 2:**
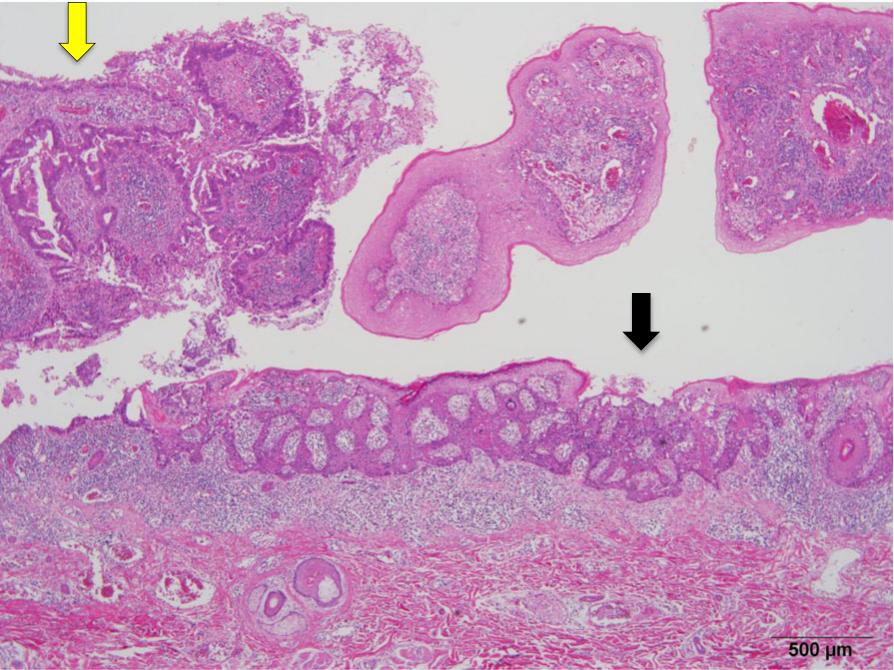
Histopathological findings revealed moderately differentiated adenocarcinoma in the tumor at the stoma site (*yellow arrow*) and pagetoid spread in the epidermis (*black arrow*)

A 53-year-old man was admitted to our hospital, complaining of a mass in his descending colon at the colostomy site. He had undergone a Hartmann’s operation to treat Hirschsprung disease in his adolescence. We diagnosed him with colon cancer at the colostomy site, which invaded the abdominal wall (Fig. 1). The descending colon, including the cancer at the stoma site, was resected, and a transverse colostomy was performed. Histopathological findings showed that the tumor was composed of moderately differentiated adenocarcinoma, and, in part, mucinous adenocarcinoma, which invaded the adjacent skin epidermis, showing pagetoid spread (Fig. 2). His cancer was pT4b (skin), pN0, cM0, stage IIC disease, according to the seventh edition of the TNM classification [3]. He refused to receive adjuvant chemotherapy but underwent follow-up examinations for 5 years.

**Fig. 3:**
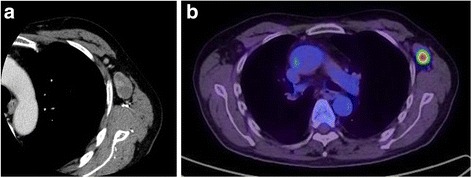
**a** Axial view of computed tomography showed a 33 × 19 mm enlarged left axillary lymph node (**b**) ^18^F-fluorodeoxyglucose positron emission tomography revealed abnormal accumulation in the left axillary fossa a maximum standard uptake value of 12.1 and no other metastatic sites

**Fig. 4:**
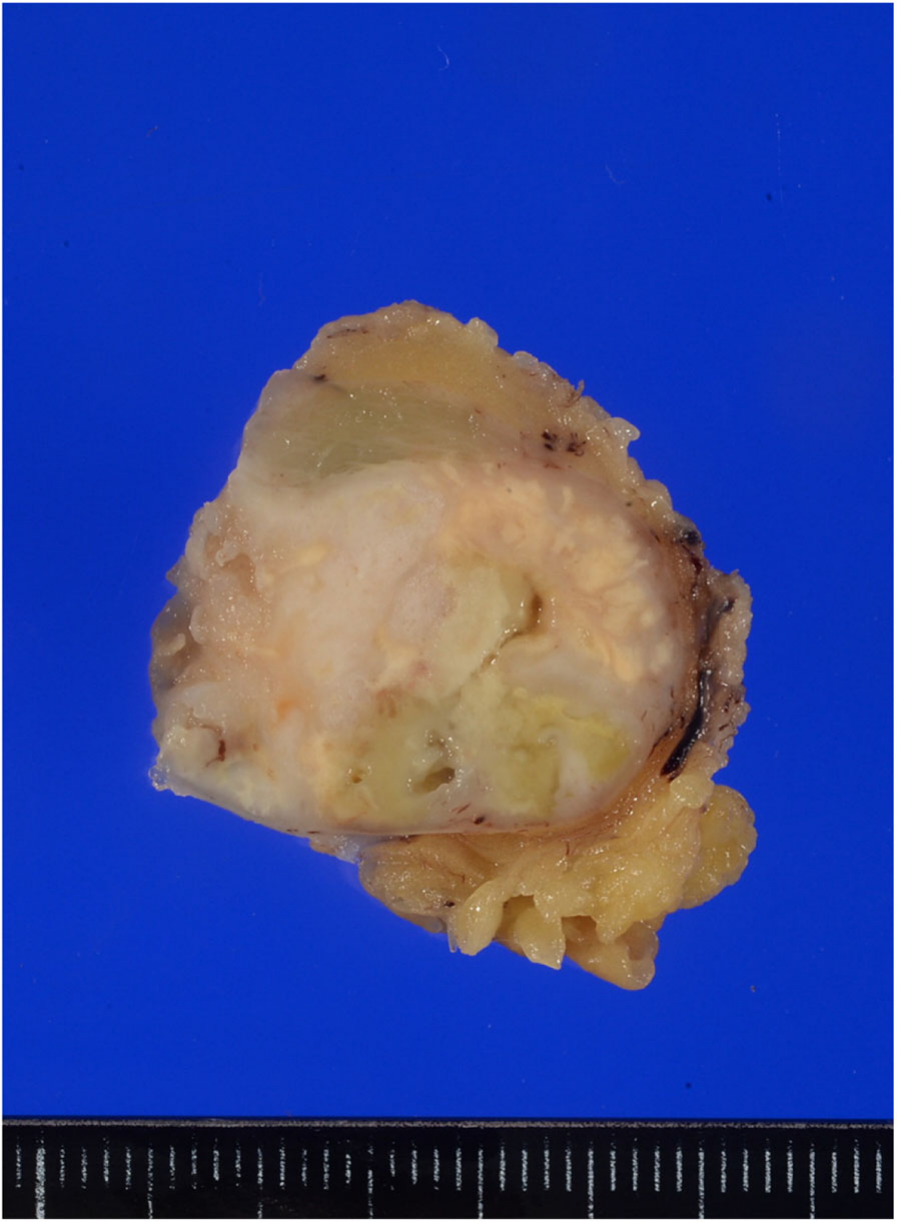
Macroscopic findings of the resected specimen revealed a white solid tumor with a necrotic tissue and a mucoid appearance

**Fig. 5:**
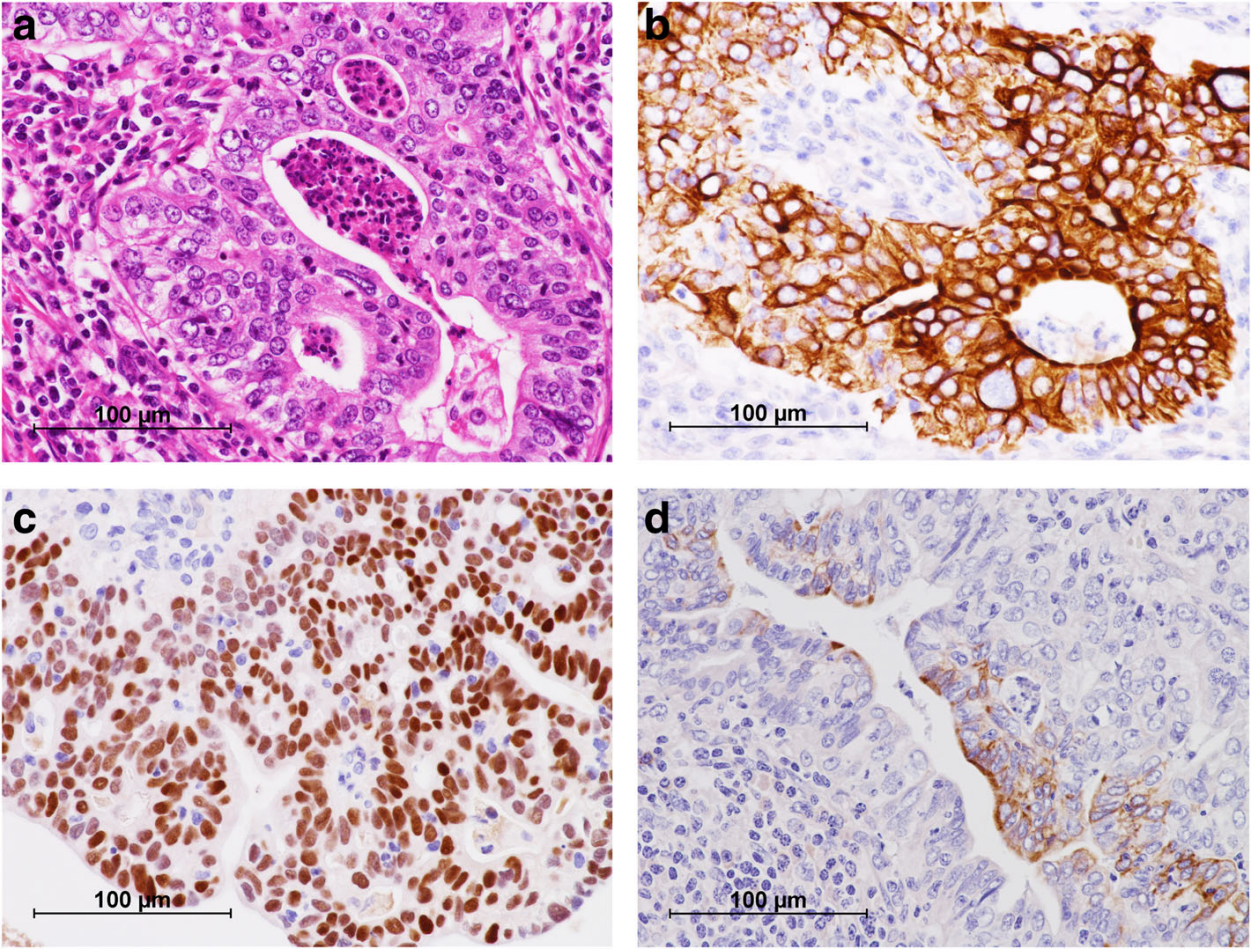
**a** Histopathological findings revealed an adenocarcinoma based on hematoxylin and eosin staining. **b**-**d** Immunohistochemically, the tumor was positive for cytokeratin 7 (**b**) and caudal-type homeobox-2 (**c**) and focally for cytokeratin 20 (**d**)

Nine years later, his carbohydrate antigen (CA) 19-9 was extremely elevated, at 157.9 IU/mL, during a health screening. On physical examination, soft and painless adenopathy with a diameter of approximately 30 mm was palpated in the left axilla. Computed tomography (CT) showed a 33 × 19 mm mass in the left axillary fossa (Fig. 3a). He underwent ^18^F-fluorodeoxyglucose (FDG) positron emission tomography/CT, and high FDG uptake was noted in the lesion (Fig. 3b). We diagnosed him with lymph node metastasis of the adenocarcinoma at the colostomy site. He underwent a curative resection of the lymph node in the left axillary fossa. Macroscopically, the central cut surface of the specimen showed a white solid tumor with necrotic tissue and a mucoid appearance (Fig. 4). The histopathological findings of the lymph node revealed that the adenocarcinoma was positive for cytokeratin 7 and caudal-type homeobox-2 and focally positive for cytokeratin 20 (Fig. 5). Based on the histological similarity between the lymph node and the resected carcinoma, we finally diagnosed the patient with left ALNM of the carcinoma at the colostomy site. His serum CA19-9 level decreased to baseline (13.8 IU/mL) in 1 month after lymph node resection. He has been receiving adjuvant capecitabine plus oxaliplatin, and he has been recurrence-free for 5 months since the lymph node resection.

### Discussion

This patient’s course revealed two important clinical facts. First, carcinoma at the colostomy site can metastasize to the axillary lymph nodes through superficial abdominal lymphatic pathways. Colon cancer metastasis to the axillary lymph nodes is rare, especially metastasis to the solitary axillary lymph node. Through our literature search, we identified five cases of colon cancer with ALNM, including only two cases with solitary ALNM [2, 4–7] (Table 1). Metastases to the axillary lymph nodes may develop either lymphatically or hematogenously [5, 7]. Lymphatic metastasis can proceed through the thoracic duct or via superficial abdominal lymphatic flow. Although common colon cancer lymphatic flow travels through the thoracic duct, Kawamura et al. demonstrated that the superficial abdominal lymphatic flow is the more important pathway in colon cancer with abdominal wall invasion, as that could lead to development of solitary ALNM [7]. The superficial lymphatic vessels of the abdominal wall below the umbilicus run downward to drain into the superficial inguinal lymph nodes, while those above the umbilicus run upward to drain into the axillary nodes [8]. Furthermore, Kim et al. reported that pagetoid spread, which is a type of intraepithelial invasion, might be related to inguinal lymph node metastasis in anal canal carcinoma [9]. Therefore, we thought that carcinoma at the colostomy site located cephalad to the umbilicus with pagetoid spread to the adjacent skin could similarly metastasize to the axillary lymph nodes through the superficial abdominal lymphatic pathway.

**Table 1:** Reported cases of axillary lymph node metastasis of colon cancer

Number	Author	Year	Age/sex	Primary tumor site	ALNM	Other metastatic lesion	Duration from primary tumor resection to ALNM	Surgery for ALNM	Therapy after surgery	Follow-up time after ALNM diagnosis (months)	Recurrence/outcome
1	Basso [4]	2007	73/M	Cecum	Left	Cervical nodes	Synchronous	+	Chemotherapy	9	(+)/dead
2	Gubitoshi [2]	2009	49/M	Left colon	Left	Intra-abdominal mass	2 years	+	RT	12	(+)/alive
3	Chieco [5]	2011	52/M	Cecum	Left	Solitary	2 years	+	FOLFIRI + RT	12	(−)/alive
4	Perin [6]	2011	49/F	Sigmoid colon	Left	Breast, lung	3 years	+	ND	16	(+)/dead
5	Kawamura [7]	2015	76/F	Cecum	Right	Solitary	2 months	+	–	ND	(−)/alive
6	Our case	2016	63/M	Descending colostomy	Left	Solitary	9 years	+	CapeOX	5	(−)/alive

*ND* not described, *RT* radiation therapy, *ALNM* axillary lymph node metastasis, *FOLFIRI* 5-fluorouracil + leucovorin + irinotecan, *CapeOX* capecitabine + oxaliplatin

Secondly, patients who undergo radical resection of solitary colon cancer ALNM followed by adjuvant chemotherapy are expected to achieve good outcomes. In general, colon cancer ALNM is regarded as a systematic disease and associated with poor prognosis in patients [4]. Some previous reports showed that the prognoses of patients who suffered from systematic lymph node metastases were poor, and systemic chemotherapies should be used to treat those patients [2, 4, 6]. In contrast, Chieco et al. reported that a patient who underwent resection of solitary cecum cancer ALNM survived for 1 year without recurrence [5]. Moreover, another report demonstrated that three patients with solitary inguinal lymph node metastasis of colon cancer survived long term (8 to 60 months) without recurrence after lymph node resection [10–12]. Another important issue is that our case did not have either regional lymph node metastasis or lymphatic invasion but developed solitary ALNM 9 years after the colon cancer resection. In the Japanese literature, non-lymphatic invasion is one of the factors of late relapse of colorectal cancer and those tumors are supposed to have less biological malignancy [13]. Thus, we considered that our case with a low malignant potential tumor had a long interval for the recurrence of solitary ALNM. Solitary axillary or inguinal lymph node metastasis of colon cancer can, therefore, be regarded as a local disease, and surgical resection seems to be an effective treatment [7, 12].

With regard to adjuvant chemotherapy for ALNM, only two cases have been reported according to our literature search [4, 5]. A patient with solitary cecum cancer ALNM received chemotherapy with FOLFIRI and radiation therapy to the left axillary fossa following lymph node resection and survived without recurrence for 12 months [5]. On the other hand, another patient with ALNM with cervical node metastasis received chemotherapy after resection of both lymph nodes and died from recurrence 9 months after surgery [4]. In the present case, the patient received adjuvant chemotherapy and has had no signs of recurrence for the 5 months after ALNM resection. Although it is unclear whether adjuvant chemotherapy improves the prognoses of patients with colon cancer ALNM, we believe that surgical resection followed by adjuvant chemotherapy would be a potent treatment for solitary colon cancer ALNM.

## Conclusions

We encountered a case of solitary left ALNM after curative resection of carcinoma at the colostomy site with pagetoid spread to the skin. This report suggests that carcinoma at the colostomy site can metastasize to the axillary lymph nodes through superficial abdominal lymphatic pathways, and surgical resection followed by adjuvant chemotherapy may be a potent treatment for solitary ALNM.

## Abbreviations

ALNM: Axillary lymph node metastasis
CT: Computed tomography CA: carbohydrate antigen
FDG: ^18^F-fluorodeoxyglucose
SUV: Standardized uptake value

## References

[1] Moritani K, Hasegawa H, Okabayashi K, Ishii Y, Endo T, Kitagawa Y (2014). Difference in the recurrence rate between right- and left-sided colon cancer: a 17-year experience at a single institution. Surg Today.

[2] Gubitosi A, Moccia G, Malinconico FA, Gilio F, Iside G, Califano UG (2009). Unusual metastasis of left colon cancer: considerations on two cases. Acta Biomed.

[3] Sobin LH, Gospodarowicz MK, Wittekind C (2009). International Union Against Cancer (UICC) TNM classification of malignant tumours.

[4] Basso L, Izzo L, Calisi E, Cavallaro G, Costi U, Ciardi A (2007). Cervical node metastasis as the first sign of cancer of the caecum. Anticancer Res.

[5] Chieco PA, Virgilio E, Mercantini P, Lorenzon L, Caterino S, Ziparo V (2011). Solitary left axillary metastasis after curative surgery for right colon cancer. ANZ J Surg.

[6] Perin T, Canzonieri V, Memeo L, Massarut S (2011). Breast metastasis of primary colon cancer with micrometastasis in the axillary sentinel node: a metastasis that metastasized. Diagn Pathol.

[7] Kawamura YJ, Kohno M, Shiga J, Asakage N, Hatano M, Okame H (2015). Right axillary lymph node metastasis of carcinoma of the cecum with histologically proven cutaneous lymphatic invasion by carcinoma cells: a case report. Surg Case Rep.

[8] Singh V (2014). Textbook of anatomy abdomen and lower limb.

[9] Kim NR, Cho HY, Baek J-H, Jeong J, Ha SY, Seok JY, et al. Rare case of anal canal signet ring cell carcinoma associated with perianal and vulvar pagetoid spread. J Pathol Transl Med. 2015. doi:10.4132/jptm.2015.08.08. 10.4132/jptm.2015.08.08 PMC4876076 26447133

[10] Pisanu A, Deplano D, Reccia I, Parodo G, Uccheddu A (2011). Unusual metachronous isolated inguinal lymph node metastasis from adenocarcinoma of the sigmoid colon. World J Surg Onc.

[11] Hara M, Takahashi H, Sato M, Takayama S, Nagasaki T, Takeyama H (2013). Curatively resected isolated inguinal lymph node metastasis from cecum cancer: report of a case. Surg Today.

[12] Iwamoto M, Kawada K, Hida K, Hasegawa S, Sakai Y (2015). Adenocarcinoma arising at a colostomy site with inguinal lymph node metastasis: report of a case. Jpn J Clin Oncol.

[13] Sato T, Suzuki M, Asaba Y, Miyake T, Tatsuyama A, Mizukami Y (2015). A case of resection of thoracic and abdominal wall metastases from colon cancer at 14 years after the first operation―a case report. Nihon Rinsho Geka Gakkai Zasshi.

